# Locus-specific control of DNA resection and suppression of subtelomeric *VSG* recombination by HAT3 in the African trypanosome

**DOI:** 10.1093/nar/gku900

**Published:** 2014-10-09

**Authors:** Lucy Glover, David Horn

**Affiliations:** Division of Biological Chemistry and Drug Discovery, College of Life Sciences, University of Dundee, Dow Street, Dundee DD1 5EH, UK

## Abstract

The African trypanosome, *Trypanosoma brucei*, is a parasitic protozoan that achieves antigenic variation through DNA-repair processes involving *Variant Surface Glycoprotein* (*VSG*) gene rearrangements at subtelomeres. Subtelomeric suppression of DNA repair operates in eukaryotes but little is known about these controls in trypanosomes. Here, we identify a trypanosome histone acetyltransferase (HAT3) and a deacetylase (SIR2rp1) required for efficient RAD51-dependent homologous recombination. HAT3 and SIR2rp1 were required for RAD51-focus assembly and disassembly, respectively, at a chromosome-internal locus and a synthetic defect indicated distinct contributions to DNA repair. Although HAT3 promoted chromosome-internal recombination, it suppressed subtelomeric *VSG* recombination, and these locus-specific effects were mediated through differential production of ssDNA by DNA resection; HAT3 promoted chromosome-internal resection but suppressed subtelomeric resection. Consistent with the resection defect, HAT3 was specifically required for the G_2_-checkpoint response at a chromosome-internal locus. HAT3 also promoted resection at a second chromosome-internal locus comprising tandem-duplicated genes. We conclude that HAT3 and SIR2rp1 can facilitate temporally distinct steps in DNA repair. HAT3 promotes ssDNA formation and recombination at chromosome-internal sites but has the opposite effect at a subtelomeric *VSG*. These locus-specific controls reveal compartmentalization of the *T. brucei* genome in terms of the DNA-damage response and suppression of antigenic variation by HAT3.

## INTRODUCTION

The African trypanosomes, *Trypanosoma brucei*, are protozoan parasites that cause sleeping sickness in humans and Nagana in livestock. The human disease is typically fatal without therapy (1–3). *T. brucei* is transmitted by tsetse flies and circulates in the mammalian host bloodstream, progressing to the fluids of the central nervous system later in the infection (4). A dense layer of a single Variant Surface Glycoprotein (VSG) covers the parasite during this stage of the life cycle and it is primarily against this VSG that an immune response is directed. Monoallelic expression of a single *VSG* gene from a subtelomeric site, and the ability of *T. brucei* to switch *VSG's*, underpins antigenic variation and immune evasion (5,6). This process of continuous antigen switching relies upon DNA recombination. Indeed, recombination is critical for the parasite to utilize its reservoir of up to 2000 subtelomeric *VSG* genes and gene fragments and to produce distinct surface coats. DNA recombination and repair mechanisms in trypanosomatids also play a major role in the emergence of drug resistance (7–9) and are also widely exploited for studies that require genetic manipulation.

Recombination is initiated by a DNA double-strand break (DSB), which most commonly occurs due to the collapse of a replication fork. In eukaryotes, chromosomal DSB repair typically involves homologous recombination (HR) or non-homologous end joining (NHEJ). In *T. brucei*, HR dominates, microhomology-mediated end joining (MMEJ) operates at a lower level and NHEJ appears to be absent (10). Fundamental to HR is resection of the 5′ ends of the DNA to generate 3′ single-stranded DNA (ssDNA) tails (2,3) on both sides of the break (11). These regions can base pair with a homologous template to promote HR (12). Indeed, it is the ssDNA that invades the duplex, donor, homologous DNA template and primes new DNA synthesis (12,13). A key enzyme required for HR is the RAD51 recombinase that coats ssDNA to form a helical ‘presynaptic’ filament that facilitates homology searching and allows for strand invasion (14,15). In *T. brucei*, RAD51-dependent and independent DSB repair reflect HR and MMEJ, respectively, and both mechanisms contribute to *VSG* switching (16,17). Spontaneous DSBs at fragile subtelomeres can trigger a *VSG* switch and the site of the break determines the outcome (16), but the controls underlying the DSB responses at these sites are not understood.

It has become clear in recent years that protein acetylation plays an important role in the DSB-repair process (18). Histone acetyltransferases and deacetylases are recruited to DSBs and modify the histones at both the damaged and template loci (19) as well as repair factors involved in HR (20). For example, Tip60 is a mammalian MYST-family histone acetyltransferase that generates binding sites and promotes accumulation of factors required for HR at the DNA-break site (21). Sirtuin-family deacetylases also promote DNA repair (22). A cycle of acetylation and deacetylation is thought to facilitate access for DNA-repair factors and then restore chromatin structure following repair, respectively (19). What has also become clear is that the DSB response must be repressed at telomeres, natural chromosomal discontinuities that are protected by a shelterin complex in mammals (23) and by a related complex in *T. brucei* (24). We reasoned that telomere structure likely impacts the subtelomeric DSB response in *T. brucei* and that this has an impact on antigenic variation.

To explore this hypothesis, we have probed the role of both acetylation and deacetylation in DSB repair in *T. brucei*, and in particular their differential impacts on DSB responses at chromosome-internal and subtelomeric *VSG* loci. Using an assay for meganuclease-induced DSB repair, we demonstrate roles for both *T. brucei* HAT3 and SIR2rp1; HAT3 is a nuclear MYST-family acetyltransferase that modifies histone H4K4 (25), and SIR2rp1 is a nuclear, sirtuin-family deacetylase previously shown to resist chemically induced DNA damage (26,27). We show that HAT3 promotes DNA resection and focal assembly of RAD51 at a chromosome-internal locus, but suppresses resection and *VSG* recombination at a subtelomeric locus. In contrast, SIR2rp1 promotes RAD51 filament disassembly at a chromosome-internal locus and has little impact on subtelomeric *VSG* recombination. These results reveal locus-specific responses to DSBs in *T. brucei* mediated by HAT3 and SIR2rp1.

## MATERIALS AND METHODS

### *T. brucei* growth and manipulation

Lister 427, MITat1.2 (clone 221a), bloodstream form cells and derivatives expressing TetR and I-SceI were described previously and were grown in HMI-11 and transformed as described (10). Cell density was determined using a haemocytometer. Limiting dilution clonogenic assays were carried out as previously described (16) using medium with or without tetracycline (1 μg/ml; Sigma). More than 98% of survivors were sensitive to puromycin (1 μg/ml; Sigma), confirming efficient disruption of the *PAC* gene during DNA break and repair.

### Plasmid construction

The pR^S^P_2110_ construct (10) was digested with XcmI prior to transfection to insert an I-SceI site within the tubulin (*TUB*) array. The pHAT3-BSD gene disruption construct was generated by replacing an *NPT* selectable marker in pHAT3-NPT (28) with a 700-bp *BSD* fragment. The other constructs used for disruption of the *SIR2rp1* gene (26) or the *HAT3* gene contained *NPT* and *BSD, PAC, HYG* or *BLE* selectable markers and were described previously (28,29). The *SIR2rpI* constructs were digested with Acc651 and SacI and the *HAT3* constructs were digested with NotI and SalI prior to transfection. *SIR2rp1* was disrupted in a *hat3*-null strain to make the double-null strain (data not shown, but see Supplementary Figure S1A). For *RAD51* gene disruption, target fragments were amplified by polymerase chain reaction (PCR) from *T. brucei* Lister 427 genomic DNA using Phusion high-fidelity DNA polymerase (New England Biolabs) in conjunction with specific primer pairs. The targets were assembled such that they flanked a *BSD* or *NPT* selectable marker. These constructs were digested with Acc651 and NotI prior to transfection. See Supplementary Table S1 for oligonucleotide sequences.

### DNA analysis

For analysis of DSB repair, slot blotting for the analysis of ssDNA was carried out as described (10). For analysis of the *TUB* locus, large DNA fragments were prepared according to standard protocols using an agarose-embedding technique. For restriction enzyme digestion, agarose blocks were incubated with enzyme for 24 h. DNA was then separated with a Bio-Rad CHEF Mapper system using an auto-algorithm set to the appropriate molecular-mass range. Gels were blotted onto nylon membranes and hybridized using standard techniques. The α*TUB* probe was a 516-bp XcmI/StuI fragment. Southern blots were processed according to standard protocols, including initial soaking in 0.25-M HCl for 15 min followed by two washes in dH_2_O. The *RFP* probe was a 687-bp HindIII/NotI fragment encompassing the full ORF, the ‘*7240’* probe was a 731-bp HindIII/XhoI coding-region fragment of Tb927.11.15600, the ‘*4250’* probe was a 2358-bp PCR product from Tb927.1.4250, the *VSG221* probe a 781-bp PstI fragment from pNEG (30) and the ‘*4530*’ probe was a 699-bp SacI fragment from pARD (31). A Typhoon TRIO phosphorimager (Amersham) was used to quantify the signals. For slot blots, percentage values for ssDNA or double-stranded DNA (dsDNA) were determined as described (10). Standard PCR reactions using Taq DNA polymerase (New England Biolabs) were used to confirm *HAT3* (oligonucleotides H31 and H34) or *SIR2rp1* disruption (oligonucleotides 3C and 5B), loss of the *RFP:PAC* substrate (oligonucleotides SceJF and Pac3Pol1), or to assess MMEJ (oligonucleotides RFP5FU and Pac3Pol1). See Supplementary Table S1 for oligonucleotide sequences.

### Western blotting and microscopy

Western blotting was carried out according to standard protocols. Rabbit anti-RAD51 primary antibodies (32) were used at 1/200. Rabbit anti-K4^acetylated^ and rabbit anti-K4^unmodified^ (25) were used at 1/2000. Blots were developed using an ECL kit (Amersham). Immunofluorescence analysis was also carried out using standard protocols as described (20). Briefly, samples were mounted in Vectashield (Vector Laboratories) containing 4, 6-diamidino-2-phenylindole (DAPI). RAD51 foci, γH2A foci and G_2_-cells were counted by both of us. Images were captured using an Eclipse E600 microscope with digital camera (Nikon) and were processed and/or deconvolved in Metamorph; all settings were identical within each data set. Fluorescein-conjugated goat anti-rabbit secondary antibody (Pierce) was used at 1:2000.

## RESULTS

### HAT3 and SIR2rp1 facilitate RAD51-dependent DNA DSB repair

**Figure 1.**
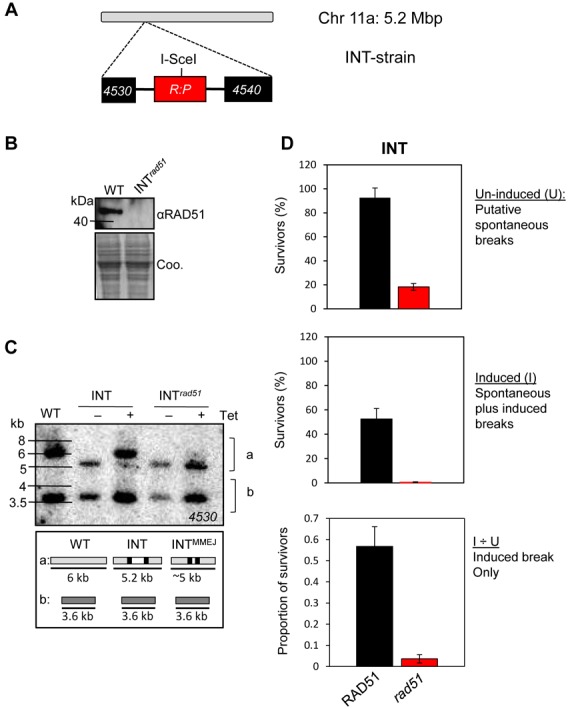
Repair at the INT locus is primarily RAD51 dependent. (**A**) The schematic illustrates the Tb927.11.4530/4540 locus on chromosome 11 in the INT strain; following insertion of an I-SceI site within a red fluorescent protein (*RFP*)–puromycin *N*-acetyltransferase (*PAC*) fusion gene (*R:P*); the I-SceI site is indicated. (**B**) Western blotting validates the INT*^rad51^* (Tb927.11.8190) null cells. Coo., Coomassie-stained gel, which serves as a loading control. (**C**) Southern blot analysis of DNA repair following an I-SceI induced break in the INT and INT*^rad51^* strains. Strains were grown in the presence or absence of tetracycline for 1 week. Genomic DNA was digested with Bsp120I and HindIII and hybridized with a ‘*4530*’ probe (see (A)). In the INT strain, the modified 5.2-kb ‘a’ allele (see panel (A)) is primarily converted back to ‘wild type’ by HR using the ‘b’ allele as a template. In the INT*^rad51^* strain, a distinct mechanism (MMEJ) generates a fragment at ∼5 kb. WT, wild-type cells; a, chromosome 11a allele; b, chromosome 11b allele. The schematic indicates the observed fragments. (**D**) Cloning efficiency in the INT strain and *rad51-*null derivative. The assay was carried out using medium with or without tetracycline to induce the DSB. Proportions of cells that recover from an induced DSB were derived by dividing the induced value by the un-induced value. Error bars, SD.

Temporal and spatial control of I-SceI meganuclease-mediated cleavage to produce a single DNA DSB has greatly facilitated the study of DSB repair in *T. brucei*. A meganuclease-mediated break at the core of chromosome 11 (Figure 1A) is predominantly repaired by HR using a single allelic template (10). Here, we refer to strains with this particular ‘chromosome-INTernal break’ as ‘INT’ strains. We first disrupted RAD51 in the INT strain (Figure 1B) and then used a Southern blot assay to monitor both the damaged and template alleles and to determine the contribution of RAD51 to HR-mediated repair at this locus (Figure 1C). We observed complete loss of the modified ‘INT’ allele and reacquisition of the repaired ‘wild-type’ allele in cells expressing RAD51, indicating efficient induction of DSBs by I-SceI and also robust repair by allelic HR (Figure 1C). In contrast, we saw no evidence for allelic HR in INT*^rad51^* null cells, indicating that RAD51 is required for allelic HR at this locus. A clonogenic assay subsequently revealed only 20% survival in the INT*^rad51^* null cells prior to I-SceI induction and less than 5% survival following induction of the break (Figure 1D). This reflects the important role for RAD51 in repairing spontaneous breaks and confirms predominant RAD51-dependent repair at this locus. Indeed, all INT*^rad51^* survivors examined (*n* = 9) repaired the break using MMEJ (33). We concluded that INT strains provided a suitable model for studies on chromosomal DNA repair and for RAD51-dependent HR in particular.

**Figure 2.**
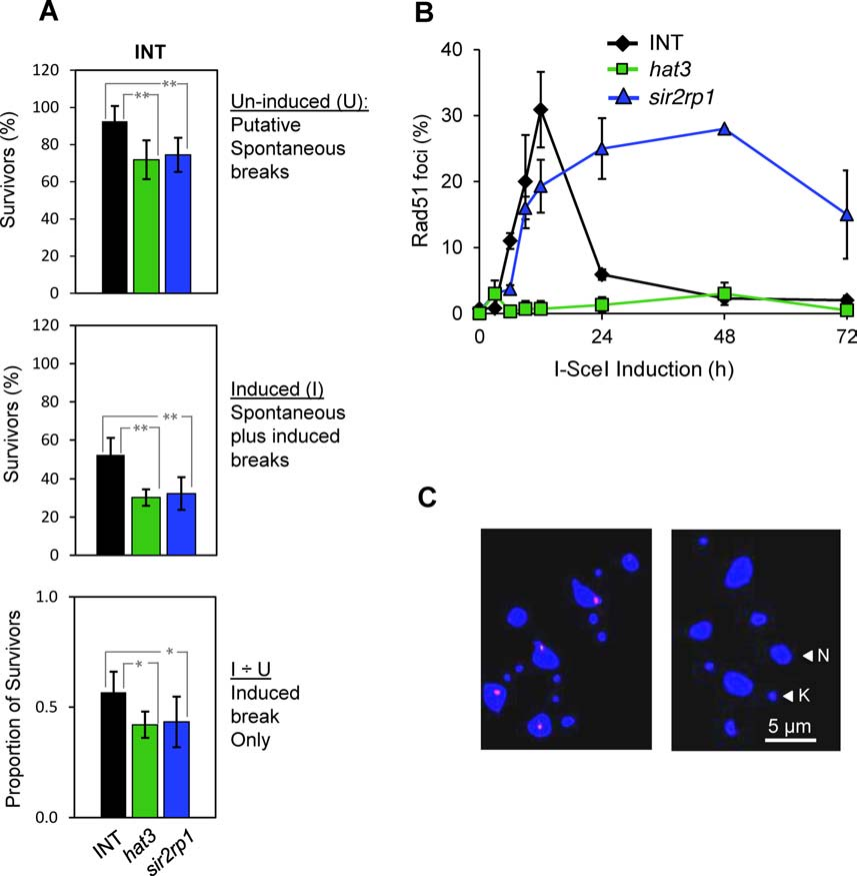
HAT3 and SIR2rp1 facilitate repair at a chromosome-internal locus. (**A**) Cloning efficiency in the INT strain. *hat3*, Tb927.10.8310-null INT strain; *sir2rp1*, Tb927.7.1690-null INT strain. A Student's *t*-test was used to derive *P* values: *P < 0.001 and **P < 0.0001. Other details are as in the legend to Figure 1D. (**B**) Monitoring of RAD51 foci. Nuclei with detectable RAD51 foci were assessed over a time course following induction of I-SceI expression. *n* = 200 at each time point. Error bars, SD. (**C**) Immunofluorescence analysis of RAD51 foci in INT (left-hand panel) and INT*^hat3^* (right-hand panel) cells following induction of I-SceI expression (+Tet for 12 h). RAD51, red; DNA counter-stained with DAPI, blue; N, nucleus; K, kinetoplast.

We suspected that acetyltransferases and deacetylases involved in DNA repair would be both localized to the nucleus and dispensable for growth. The acetyltransferase, HAT3, and the deacetylase, SIR2rp1, both met these criteria and these were, therefore, selected for analysis using the chromosomal ‘INT’ DNA-repair assay. We disrupted either *HAT3* or *SIR2rp1* in the INT strain (Supplementary Figure S1A and B) and used a clonogenic assay to assess the capacity for recovery from DNA damage in these cells. Notably, the INT*^hat3^* and INT*^sir2rp1^* strains displayed reduced survival even prior to induction of the DSB (Figure 2A). We speculated that this might reflect a role for both of these proteins in the repair of spontaneous breaks. Following induction of the DSB, ∼50% of the INT cells fail to survive, as reported previously (10). Importantly though, both INT*^hat3^* and INT*^sir2rp1^* cells displayed a further, significant reduction in survival following induction of the break, indicating a further defect in DSB repair at this chromosome-internal site (Figure 2A). Although the efficiency of DSB repair was reduced, both allelic HR and MMEJ continued to operate in both *hat3* and *sir2rp1*-null strains (Supplementary Figure S1C and D).

Following a meganuclease-induced DNA break, a diffusible nuclear pool of RAD51 relocalizes to sites of damage to form sub-nuclear foci that can be detected by microscopy (10). Since DNA repair at the INT locus is primarily RAD51 dependent (Figure 1D), we examined the impact of HAT3 and SIR2rp1 on the focal accumulation of RAD51 during DSB repair. Upon induction of the DSB in INT cells, the proportion of cells with RAD51 foci increased rapidly from ∼0% to 30% within 12 h, and declined thereafter (Figure 2B). We observed striking and contrasting phenotypes in the *hat3* and *sir2rp1*-null strains. INT*^hat3^* cells fail to assemble RAD51 foci whilst INT*^sir2rp1^* cells fail to disassemble RAD51 foci (Figure 2B). Figure 2C shows the foci as observed in control cells, compared to the absence of foci in INT*^hat3^* cells; the persistent foci seen in INT*^sir2rp1^* cells were indistinguishable from those seen in controls. These results reveal roles for HAT3 and SIR2rp1 in the assembly and disassembly of RAD51 foci and in DSB repair.

### A synthetic defect in *hat3-sir2rp1* double-null *T. brucei*

**Figure 3.**
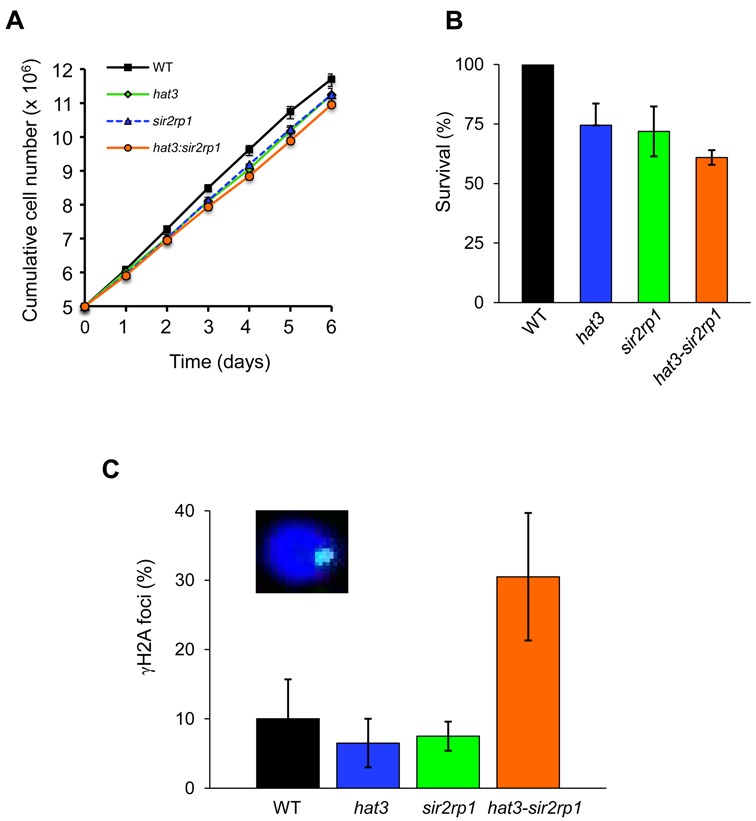
A synthetic defect in *hat3-sir2rp1* double-null *T. brucei.* (**A**) Growth of wild-type, *hat3, sir2rp1* and *hat3:sir2rp1* double-null strains. Error bars, SD. (**B**) Cloning efficiency of the wild-type (WT) *hat3, sir2rp1* and *hat3-sir2rp1* double-null strains. Error bars, SD. (**C**) Monitoring of γH2A foci in the *hat3, sir2rp1* and *hat3-sir2rp1* double-null strains. Nuclei with detectable γH2A foci were counted, *n* = 200 for each sample. Error bars, SD. Inset: immunofluorescence image of a γH2A focus (green); DNA was counter-stained with DAPI, blue.

Failure to assemble RAD51 foci and failure to disassemble RAD51 foci in the *hat3* and *sir2rp1*-null strains, respectively, suggests distinct roles for these two proteins in DNA repair. To further explore this hypothesis, we assembled a double-null *T. brucei* strain lacking both HAT3 and SIR2rp1. We observed a moderate growth defect for either *hat3*-null or *sir2rp1*-null strains and a further, moderately enhanced, growth defect in the double null (Figure 3A). The *hat3-sir2rp1* double-null strain also displayed moderately reduced survival in a clonogenic assay relative to either single-null strain (Figure 3B). Notably though, we observed a substantial increase in the proportion of double-null cells with γH2A foci relative to either single-null strain (Figure 3C); phosphorylation of histone H2A and the formation of γH2A are an early marker for DNA damage in *T. brucei* (34). This synthetic defect supports the hypothesis that these two proteins play distinct roles in DNA repair.

### HAT3 suppresses subtelomeric *VSG* recombination and antigenic variation

**Figure 4.**
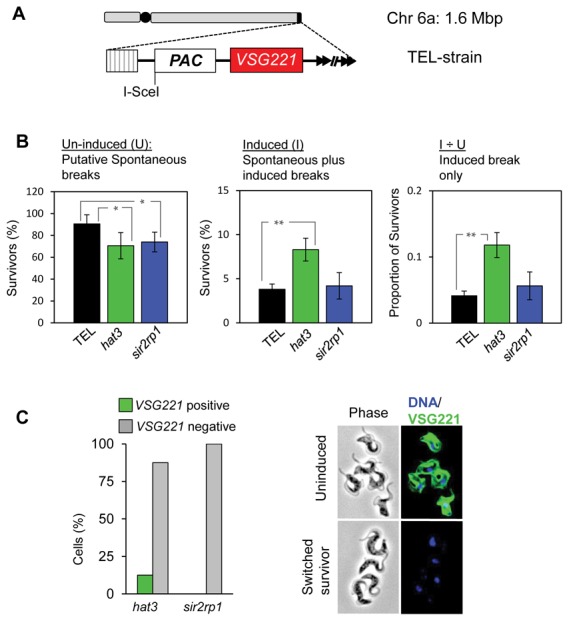
HAT3 suppresses subtelomeric *VSG* recombination. (**A**) The schematic illustrates the subtelomeric *VSG221* locus on chromosome 6a in the TEL strain after insertion of the *^Sce^PAC* cassette; the I-SceI site is indicated; black circle, centromere; striped box, 70-bp repeats; arrowheads, telomeric repeats. (**B**) Cloning efficiency in the TEL strain and *hat3* and *sir2rp1*-null derivatives. *P < 0.01 and **P < 0.001. Other details are as in the legend to Figure 1D. (**C**) Survivors from the clonogenic assays were scored by VSG221 immunofluorescence microscopy. *hat3*,*n* = 8; *sir2rp1*,*n* = 25. The VSG221 immunofluorescence images show an example of a switched survivor. DNA was counter-stained with DAPI.

Although a DNA break close to the active subtelomeric *VSG* gene serves as a trigger for RAD51-dependent *VSG* switching (16,35), no increase in RAD51 foci is observed during repair at this locus (16). Since HAT3 and SIR2rp1 control RAD51 assembly and disassembly, respectively, at the INT locus, we examined their contribution to repair at the *VSG* locus using a genetic approach. The *HAT3* and *SIR2rp1* genes were disrupted in a ‘TELomeric’ or ‘TEL’-strain (Supplementary Figure S1A and B), with an I-SceI site engineered upstream of the active *VSG* gene (Figure 4A). Similar to what we observed in the INT strain (Figure 2A), both TEL*^hat3^* and TEL*^sir2rp1^*-null strains displayed reduced survival in a clonogenic assay, again likely due to defective repair of spontaneous breaks (Figure 4B). In contrast to what we observed in the INT strain (Figure 2A), however, neither null TEL strain displayed further reduced survival following induction of a DSB at the *VSG* locus (Figure 4B). In fact, we observed a significant increase in survival in TEL*^hat3^* null cells.

Survival typically requires replacement of the active *VSG* with a different *VSG* following an induced DSB in the TEL strain (16). Cloned survivors from TEL*^hat3^* and TEL*^sir2rp1^* strains were assessed for VSG switching using immunofluorescence analysis. More than 99% of cells expressed VSG221 prior to induction of the break whilst almost all (but one) of the survivors (*n* = 33) had switched off VSG221 expression (Figure 4C). These results confirm efficient induction of DSBs by I-SceI in both TEL*^hat3^* and TEL*^sir2rp1^* strains and indicate continued subtelomeric repair and *VSG* replacement in the absence of either HAT3 or SIR2rp1. We conclude that HAT3, in striking contrast to its role in RAD51-focus assembly and repair at a chromosome-internal locus, suppresses antigenic variation through *VSG* recombination at a subtelomeric locus.

### Locus-dependent control of ssDNA accumulation by HAT3

**Figure 5.**
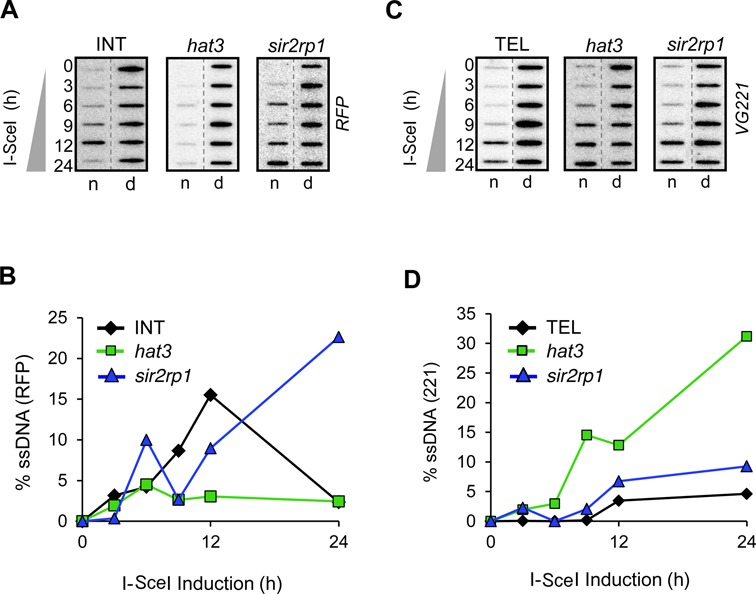
Locus-dependent control of DNA resection by HAT3. Accumulation of ssDNA adjacent to a DSB was monitored by slot-blot analysis in the INT (**A**) and TEL (**C**) strains. Genomic DNA was extracted at the times indicated following I-SceI induction. Ninety percent of each sample was ‘native’ (n; where a hybridization signal with native DNA indicates the presence of ssDNA) and the remainder was denatured (d). The probes used on each blot are indicated on the right (see the schematic maps in Figures 1A and 4A). ssDNA signals from the INT (**B**) and TEL (**D**) strains were quantified by phosphorimager analysis as previously described (10).

We next sought the mechanism by which HAT3 and SIR2rp1 differentially control DNA repair at chromosome-internal and subtelomeric loci. Since DNA resection to generate ssDNA is required for RAD51-focus assembly at the site of the break, we monitored the kinetics of ssDNA accumulation over time adjacent to breaks in the INT and TEL strains (Figure 5). In INT cells, ssDNA accumulates adjacent to a break at the chromosome-internal locus 12 h after induction and declines thereafter (Figure 5A and B), as also described previously (10). In contrast, and consistent with the defects in RAD51-focal assembly and disassembly (Figure 2B and C), ssDNA fails to accumulate in INT*^hat3^* null cells, and persists in INT*^sir2rp1^* null cells (Figure 5A and B).

Analysis of ssDNA following a break at the TEL locus confirmed locus-specific control of the DNA-damage response by HAT3. In this case, we observed little difference between TEL cells and TEL*^sir2rp1^* null cells, but increased ssDNA accumulation in TEL*^hat3^* null cells (Figure 5C and D). Thus, HAT3 promotes DNA resection and RAD51 assembly at a chromosome-internal locus but suppresses DNA resection and *VSG* recombination at the subtelomeric *VSG* locus. No increase in RAD51 foci was detected in TEL*^hat3^* cells, despite the accumulation of *VSG221* ssDNA (data not shown).

### HAT3 and SIR2rp1 facilitate DNA resection at a multi-copy chromosome-internal locus

**Figure 6.**
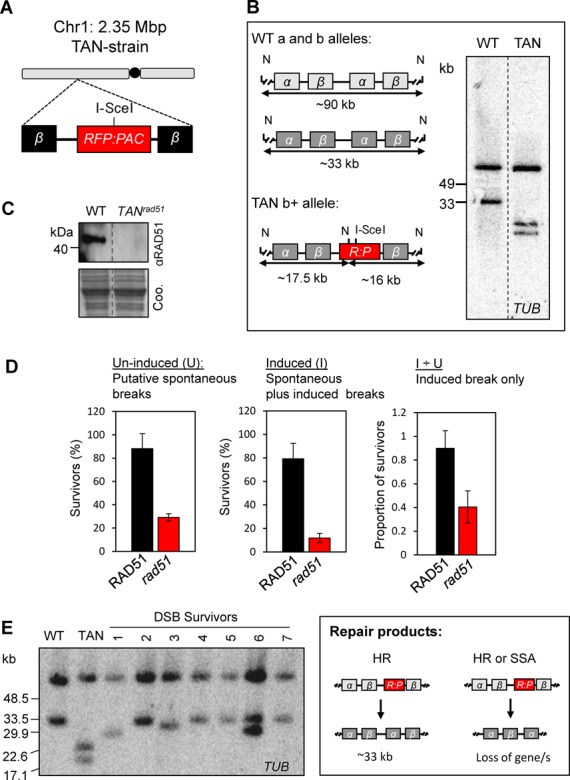
DSB repair within the *tubulin/*TAN gene array (Tb927.1.2340-2400). (**A**) The schematic illustrates the *TUB* locus on chromosome 1 after replacement of an α-*TUB* gene with the *RFP*:*PAC* cassette. The I-SceI recognition site is indicated; black circle, centromere; *β, β-TUB* genes. (**B**) The schematic illustrates analysis of the *TUB* alleles on chromosome 1; the polymorphic wild-type (WT) a and b alleles (37) and the b allele after insertion of the *R^s^P* cassette (TAN, b+) are shown. The approximate sizes of the NcoI (N) fragments expected on Southern blots are indicated below the maps. For Southern blotting, genomic DNA was digested with *Nco*1 and subjected to pulsed-field gel electrophoresis. Bands corresponding to the WT and b+ alleles are present. (**C**) Western blotting validates the TAN*^rad51^* null cells. Coo., Coomassie-stained gel, which serves as a loading control. (**D**) Cloning efficiency in the TAN*^rad51^*-null strain. Other details are as in the legend to Figure 1D. (**E**) DSB-repair survivors were cloned in medium containing tetracycline and genomic DNA was analysed by Southern blotting as described in (B) above. Bands corresponding to the ‘WT’-b and truncated b alleles are recovered. The schematic illustrates repair, which we predict is via either allelic homologous recombination (survivors 2, 4 and 5–7) or single-strand annealing (survivors 1, 3 and 6).

The locus-specific functions of HAT3 may be explained by the large number of potential templates available for subtelomeric repair or by differences between subtelomeres and other chromosomal loci. To begin to address these alternative hypotheses, we established strains for the analysis of DNA repair at a multi-copy but chromosome-internal locus. The *T. brucei* genome, in the context of widespread constitutive polycistronic transcription, employs tandem gene duplication to increase expression (36), as exemplified by the tubulin gene array on *T. brucei* chromosome 1 (Figure 6A). To generate the ‘TANdem’ or ‘TAN’ strain, an I-SceI cleavage site was targeted to one of ∼30 *α/β tubulin* repeats (37,38). Southern blot analysis indicated an I-SceI site inserted within the shorter gene array (Figure 6B).

As was the case at the TEL locus, we detected no increase in RAD51 foci following a DSB at the *tubulin*/TAN locus (data not shown). To determine the contribution of RAD51 to repair at this locus, we generated TAN*^rad51^* null cells (Figure 6C). A clonogenic assay revealed remarkably efficient repair following a DSB at this locus (Figure 6D). Once again though (see Figure 1D), we observed a major RAD51-associated defect in the repair of spontaneous breaks (Figure 6D). Consistent with approximately equal repair by RAD51-dependent allelic HR or inter-chromosomal single-strand annealing (SSA), approximately half of the induced breaks appeared to be successfully repaired in the absence of RAD51 (Figure 6D); SSA is typically RAD51 independent (39) and can operate when a break occurs between two repeated sequences oriented in the same direction. During SSA, single-stranded regions extend to the repeated sequences such that the complementary strands can anneal to each other, leading to repair and the loss of one or more of the repeats. Among seven cloned DSB survivors, four appeared to reflect allelic HR, regenerating the short array, two revealed deletions and one revealed a mixture of these outcomes; the deletions may arise due to HR but most likely arise due to SSA (Figure 6E). These results indicate highly efficient induction of I-SceI-mediated breaks and highly efficient repair of these breaks at the *tubulin*/TAN locus.

**Figure 7.**
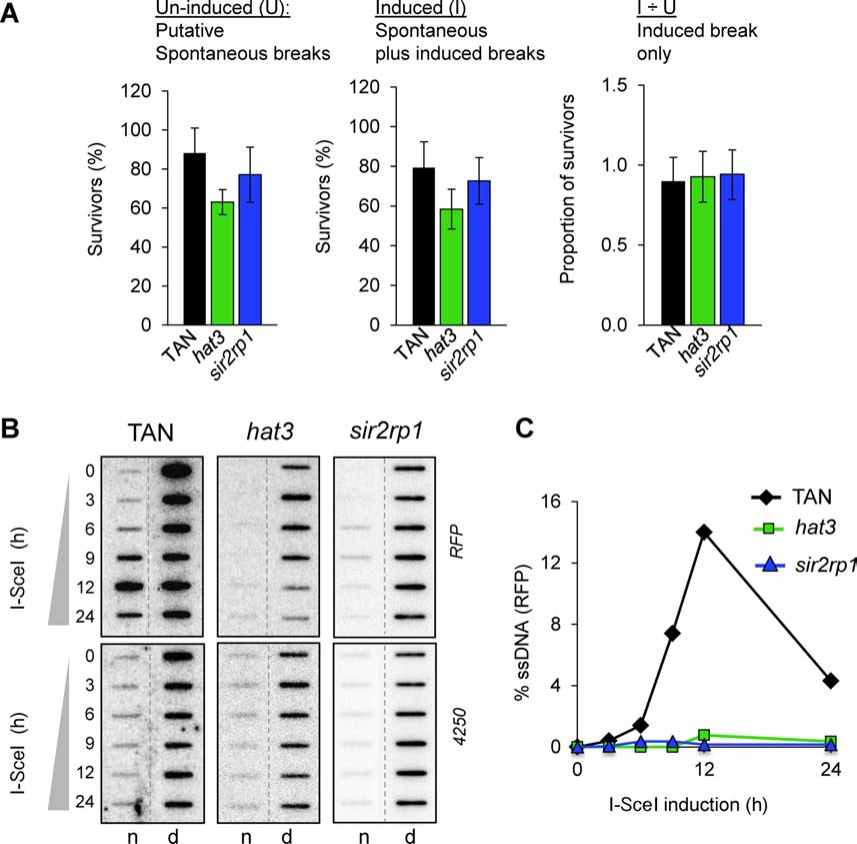
HAT3 and SIR2rp1 facilitate DNA resection at a chromosome-internal tandem gene array. (**A**) Cloning efficiency in the TAN strain, *hat3*-null and *sir2rp1*-null derivatives. Other details are as in the legend to Figure 1D. (**B/C**) Accumulation of ssDNA adjacent to the DSB was monitored as described in the legend to Figure 5A/C. The loading control was prepared using a ‘4250’ probe also from chr. 1 (see the Materials and Methods section). (C) Signals derived from the TAN strains were quantified by phosphorimager analysis as described (10).

To address the role of HAT3 or SIR2rp1 in repair at the *tubulin/*TAN locus, we disrupted the *HAT3* or the *SIR2rp1* genes in the TAN strain (Supplementary Figure S1A). In clonogenic assays, neither protein had a detectable effect on the repair of induced breaks (Figure 7A). As seen following DNA breaks at other sites, ssDNA adjacent to the break was most abundant 12 h following induction in the TAN strain and declined thereafter. The *hat3* defect at the *tubulin* locus was similar to the defect at the INT locus; resection was again severely compromised (Figure 7B and C). Notably though, we also observed a resection defect in the *sir2rp1*-null strains (Figure 7B and C), suggesting an earlier role for SIR2rp1 at the TAN locus compared to the role observed at the INT locus. In the case of the TAN*^hat3^*-null strain, the *RFP* signal was rapidly depleted compared to the control or TAN*^sir2rp1^*-null strains. This suggested more rapid loss of dsDNA adjacent to the break (Figure 7B; compare the 12- and 24-h time points, d-column) and a PCR-based assay suggested that this was in fact due to more rapid cleavage by I-SceI in the TAN*^hat3^-*null strain (Supplementary Figure S1E). These results indicate that both HAT3 and SIR2rp1 facilitate DNA resection at a tandem-arrayed chromosome-internal locus, that only HAT3 facilitates DNA resection at a ‘single-copy’ locus and that neither protein is required for DNA resection at the subtelomeric *VSG* locus. We can also conclude that DNA repair continues to operate effectively at the TAN locus, despite the resection defect; residual resection must be sufficient when multiple adjacent templates are available for SSA and/or when multiple allelic templates are available for HR.

### Locus-dependent control of the G_2_ DNA-damage checkpoint response

**Figure 8.**
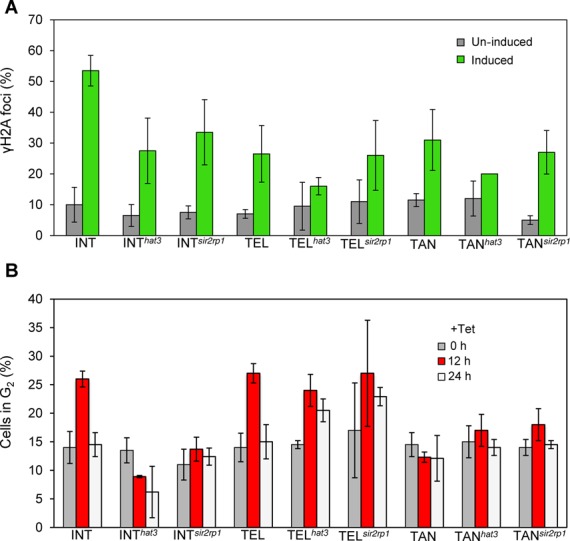
Impact of HAT3 or SIR2rp1 on γH2A foci and the G_2_ DNA-damage checkpoint response. (**A**) Monitoring of γH2A foci in the INT, TEL or TAN strains with or without *HAT3* or *SIR2rp1*. Nuclei with detectable γH2A foci were counted 12 h after I-SceI induction. See the legend to Figure 3C for other details. (**B**) Monitoring of nuclei in the G_2_-phase of the cell cycle in the INT, TEL or TAN strains with or without *HAT3* or *SIR2rp1.* G_2_ cells contain a single nucleus and two kinetoplasts. Counts were taken 0, 12 and 24 h after I-SceI induction (*n* ≥ 90 cells at each time point).

The DSB response is characterized by a cascade of events. Following the formation of γH2A foci (34), DNA resection and the assembly of RAD51 foci can culminate in an arrest at the G_2_-phase of the cell cycle (10,16). There is also evidence that histone acetylation and γH2A act cooperatively in the DNA-damage response in human cells (40). To explore the relationship amongst different steps in the *T. brucei* repair cascade, we assessed the accumulation of γH2A and G_2_-checkpoint function in response to breaks at chromosome-internal and subtelomeric loci. Cells with γH2A foci are present at a low level in unperturbed cells and increased ∼5-fold in cells with induced breaks (Figure 8A). We observed ∼2-fold reduction in detectable γH2A foci following DSB induction at all three loci in cells lacking HAT3 (Figure 8A). We saw a similar effect in cells lacking SIR2rp1, but this appeared to be restricted to the INT locus in this case (Figure 8A). Thus, the formation of γH2A foci is moderately affected by HAT3 function and, at a chromosome-internal locus, also by SIR2rp1 function.

For cell-cycle analysis, DAPI-stained nuclear and mitochondrial (kinetoplast) DNA in *T. brucei* can be used as cytological markers. Cells with one nucleus and two distinct kinetoplasts (1N2K) are defined as being in the G_2_-phase. Examination of the G_2_-checkpoint revealed responses following breaks at the INT and TEL loci (Figure 8B). Surprisingly, we see no G_2_-checkpoint response in TAN cells (Figure 8B), despite robust resection (Figure 7B); resection and G_2_-checkpoint activation are typically thought to be part of the canonical DNA-damage response in eukaryotes (41). Consistent with impacts on the efficiency of DNA repair, the G_2_-checkpoint response was ablated specifically following a break at the chromosome-internal locus in cells lacking either HAT3 or SIR2rp1 (Figure 8B, compare INT and TEL strains). Thus, SIR2rp1 promotes efficient γH2A-focus assembly, RAD51-focus disassembly and efficient DNA repair at a ‘single-copy’ locus but promotes DNA resection at a tandem-arrayed locus. In contrast, HAT3 promotes efficient γH2A-focus assembly, DNA resection, RAD51-focus assembly and efficient DNA repair at a chromosome-internal, ‘single-copy’ locus but suppresses DNA resection and DNA repair at a subtelomeric *VSG* locus.

## DISCUSSION

Although loss of a *T. brucei* telomere fails to trigger a DSB response (42), a subtelomeric break can trigger *VSG* recombination and antigenic variation (16). These results suggest a gradient of DSB-response suppression starting at the telomere and this may reflect the particular features of chromatin structure that protect the ends of chromosomes (23,24). Since the major mechanism of antigenic variation in *T. brucei* involves *VSG* recombination at subtelomeres, these structures have an important impact on the capacity for immune evasion. To further explore locus-specific responses to DSBs and the impact on antigenic variation, we identified chromatin modifiers with a role in DNA repair and probed the impact of these factors on DSB repair at two chromosome-internal sites and one subtelomeric site. We demonstrate distinct roles for HAT3 histone acetyltransferase and the SIR2rp1 deacetylase in DNA resection and DSB repair. HAT3 has opposite impacts on DNA repair at chromosome-internal and subtelomeric loci. The results suggest that protein acetylation by HAT3 and deacetylation by SIR2rp1 are important for efficient HR at a chromosome-internal locus. In contrast, HAT3 specifically suppresses DNA resection and *VSG* recombination at a subtelomeric locus.

RAD51-dependent HR dominates DSB repair at a chromosome-internal locus in *T. brucei* (10). Consistent with the defects in resection and RAD51-focus formation at this site, HAT3 was specifically required for the G_2_-checkpoint response. Mammalian Tip60, another MYST-family histone acetyltransferase, is required to generate γH2A foci (43), and we also observe compromised γH2A-focus formation in the absence of HAT3. RAD51-dependent HR also makes an important contribution to DSB repair at a subtelomeric *VSG* locus (16). However, although RAD51 foci are readily visible following a DSB at the chromosome-internal site, and our genetic analyses indicate ∼50% RAD51-dependent repair at either a subtelomere (16) or within a tandem array (the current work), RAD51 foci are not visible following a DSB at these latter two sites. This suggests that less RAD51 is required for homology searching and repair in these cases, possibly due to the large number of alternative templates available for repair. Indeed, efficient repair operated independent of HAT3 and was suppressed by HAT3, respectively, at the tandem-arrayed locus and at the *VSG* locus. These results do not reveal whether the repair suppressed by HAT3 at the *VSG* locus is RAD51 dependent or independent, but they do suggest that the availability of multiple repair templates compensates for a HAT3 defect.

DSB repair requires chromatin remodelling but the underlying mechanisms remain only partially characterized. Remodelling in the region surrounding the break is thought to prime the DSB and facilitate access to the DNA-end-resection machinery. It is thought that the ssDNA tails then serve to activate the DNA-damage response. Following recombination, further remodelling is then thought to restore the original chromatin structure; this is known as the ‘Prime, Repair, Restore’ model (44). Specifically, histone acetylation by Tip60 (21) or MOF (45,46) in mammalian cells or Esa1 in yeast (47) promotes accumulation of factors required for recombination. Sirtuin-family deacetylases also promote DNA repair (22) and, potentially analogous to the situation we report in *T. brucei*, RAD51 focus disassembly is delayed by SIRT1 knockdown (48) or chemical inhibition of sirtuins (49) in mammalian cells. Indeed, SIRT1 physically interacts with and negatively regulates Tip60 in these cells (48). Since Tip60, Esa1 and HAT3 are all MYST-family histone acetyltransferase, our results suggest that cycling of acetylation and deacetylation by MYST acetyltransferases and sirtuins may play an important and conserved role in genome integrity from trypanosomes to humans.

Although Tip60-dependent histone acetylation is one of the best characterized responses to a DSB in mammalian cells (18), this acetyltransferase is also required for the activation of the ATM (ataxia telangiectasia mutated) kinase (50,51), an early DNA-damage sensor (14). Thus, HAT3 may mediate its effects on DNA repair in *T. brucei* through acetylation of histone H4K4 (25), and/or through the acetylation of other factors, or even potentially by an indirect mechanism. Sirtuins also deacetylate non-histone repair factors (51,52) so SIR2rp1 could equally mediate its effects through deacetylation of histones or other non-histone substrates. Indeed, SIR2rp1 does not appear to deacetylate histone H4K4 (25) and the synthetic defect we report in *hat3-sir2rp1* double-null cells suggests that these two proteins mediate their distinct impacts on DNA repair by modifying distinct substrates. Several sirtuins, including *T. brucei* SIR2rp1 also exhibit ADP ribosyltransferase activity (27). The locus-specific effects that we observe in response to DNA damage are also consistent with distinct roles for HAT3 and SIR2rp1.

The prime, repair, restore model for DNA repair invokes a strict temporal order of post-translation modification. For example, acetylation allows repair factors to negotiate the barrier presented by chromatin, and deacetylation switches off the DNA-damage response and allows cells to resume the cell-cycle following repair. Our results are entirely consistent with this model. The HAT3 defect impacts γH2A-focus assembly, one of the earliest steps in the DNA-damage response, and also leads to almost complete failure to resect DNA, assemble RAD51 foci or trigger a G_2_-checkpoint. The known modification mediated by HAT3 is constitutive acetylation of histone H4K4 (25). Post-damage exposure of constitutive chromatin modification has been proposed in DNA damage signalling (53), and we also suggest a role for cell-cycle-regulated exposure in the case of acetylated H4K4; this modification is specifically inaccessible to antibodies in fixed *T. brucei* cells in the G_1_-phase of the cell cycle (25). Thus, access to acetylated H4K4 could delay the DNA-damage response until the S- and G_2_-phases of the cell cycle. Later in a repair cycle, strand invasion and new DNA synthesis are thought to precede disassembly of RAD51 foci (54). Telomeric sirtuins in other cell types relocalize to sites of damage to mediate their effects (55,56). Our results indicate a role in disassembly of RAD51 foci at chromosome-internal sites and, since SIR2rp1 functions in telomeric silencing in *T. brucei* (26), we suggest that relocalization also operates in *T. brucei* to mediate this effect.

We show that HAT3 is required for efficient DNA resection, focal assembly of RAD51, G_2_-checkpoint activation and DNA repair. In contrast, HAT3 suppressed DNA resection and recombination at a subtelomeric locus. Thus, we see a correlation between DNA resection and the efficiency of DNA repair that is locus specific and dependent upon the action of HAT3. We also describe a locus-specific impact for SIR2rp1 on DNA repair, with little impact on subtelomeres in this case. The locus dependence we observe in *T. brucei* is important for antigenic variation, which depends upon *VSG* recombination at subtelomeres. Indeed, the vast majority of *VSG* genes are located in subtelomeric arrays and many are flanked by 70-bp repeats (57) and telomeric repeats, which range in both sequence and size. The 70-bp repeats create sequence homology that defines the boundary of the duplicative transposition of *VSG* genes during recombination (58). Given that donor *VSGs* are typically thought to be within heterochromatin, chromatin remodelling could be required to allow invasion of a RAD51 synaptic filament. Indeed, chromatin remodelling facilitates recombination within heterochromatin in *Saccharomyces cerevisiae* (59) so we must consider the impact of chromatin modifiers at both the damaged and donor sites during HR. As well as *T. brucei* HAT3, other factors, such as the type-IA topoisomerase, TOPO3α (60), the telomere-interacting factor, TIF2 (61) and long telomeres (62), also suppress *VSG* recombination.

An understanding of the control of DNA resection and resolution of recombination intermediates has remained elusive. The approach taken here has allowed us to begin to dissect the role of HAT3 and SIR2rp1 in DSB repair at three different chromosomal loci in *T. brucei*, two chromosome-internal and one subtelomeric. Our data suggest a cycle of protein acetylation and deacetylation contributing to a DSB response that facilitates repair at a chromosome-internal locus. Several acetyltransferases and deacetylases impact the DNA-repair process in eukaryotes and we now show that a pair of these enzymes controls the cycle of DNA resection and RAD51 assembly and disassembly, respectively, in *T. brucei*. Our finding that HAT3 suppresses subtelomeric recombination and antigenic variation also reveals compartmentalization of the *T. brucei* genome in terms of DNA repair.

## SUPPLEMENTARY DATA

Supplementary Data are available at NAR Online.

SUPPLEMENTARY DATA
