# Supplementary material for: Locus-specific control of DNA resection and suppression of subtelomeric VSG recombination by HAT3 in the African trypanosome
Source: Nucleic Acids Res. 2014 Oct 9;42(20):12600–13. doi: 10.1093/nar/gku900 (PMC4227765; doi:10.1093/nar/gku900)
Supplement: SUPPLEMENTARY DATA [file supp_42_20_12600__index.html]

Locus-specific control of DNA resection and suppression of subtelomeric VSG recombination by HAT3 in the African trypanosome — Locus-specific control of DNA resection and suppression of subtelomeric VSG recombination by HAT3 in the African trypanosome — SUPPLEMENTARY DATA 

# Locus-specific control of DNA resection and suppression of subtelomeric *VSG* recombination by HAT3 in the African trypanosome

## SUPPLEMENTARY DATA

**Files in this Data Supplement:**

- SUPPLEMENTARY DATA
