# Supplementary material for: Locus-specific control of DNA resection and suppression of subtelomeric VSG recombination by HAT3 in the African trypanosome
Source: Nucleic Acids Res. 2014 Oct 9;42(20):12600–13. doi: 10.1093/nar/gku900 (PMC4227765; doi:10.1093/nar/gku900)
Supplement: SUPPLEMENTARY DATA [file supp_gku900_nar-01483-x-2014-File003.pdf]

Locus-specific control of DNA break processing and suppression of subtelomeric *VSG* recombination by HAT3 in the African trypanosome

Lucy Glover and David Horn

Division of Biological Chemistry & Drug Discovery, College of Life Sciences, University of Dundee, Dow Street, Dundee DD1 5EH, UK.

Supplementary Figure S1

Supplementary Table S1

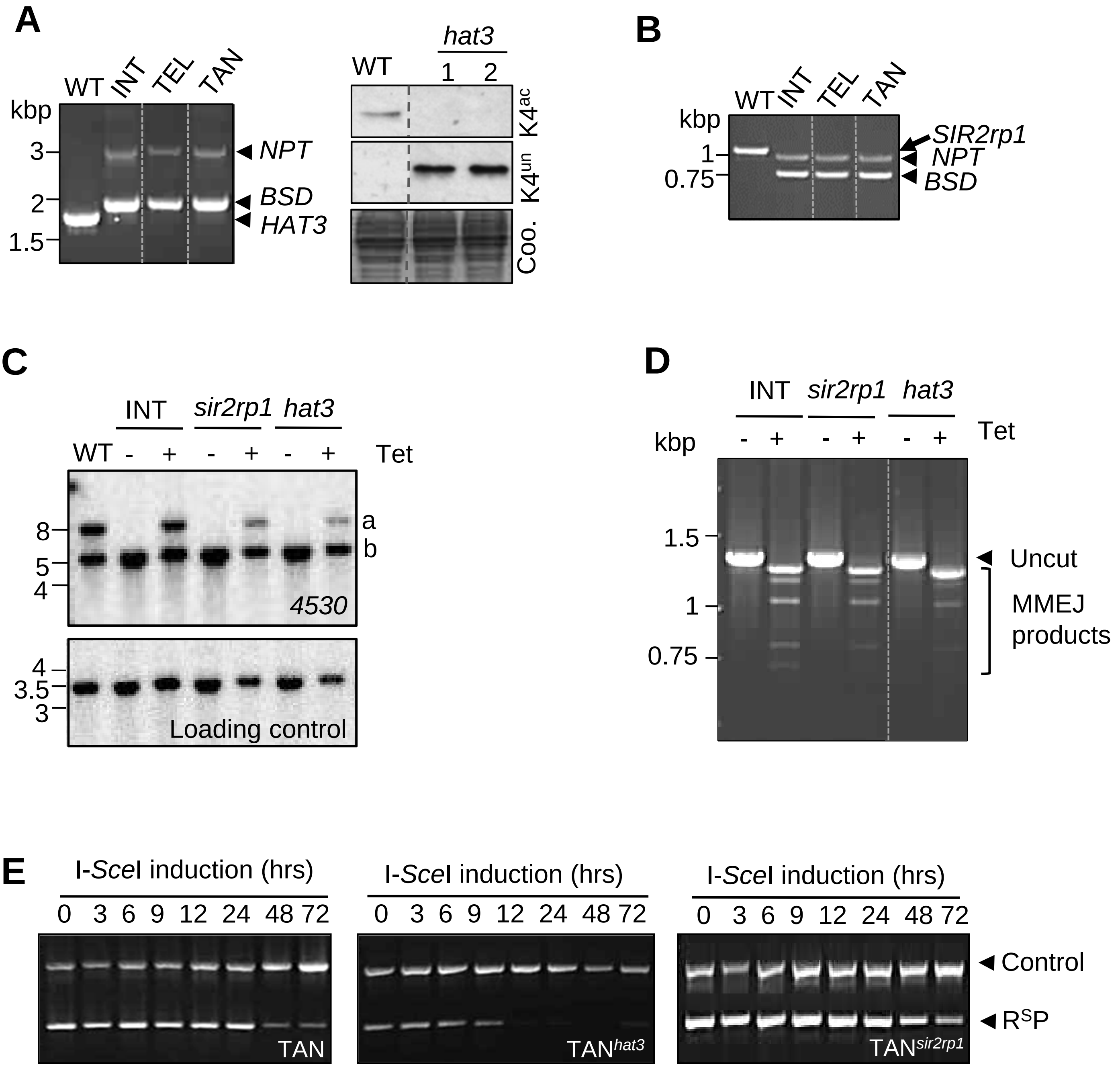

Supplementary Figure S1. Validation and assessment of *hat3* and *sir2rp1*-null strains. (**A**) A PCR-based assay (left-hand side) indicates disruption of native *HAT3* and replacement with *NPT* and *BSD* selectable marker cassettes in the INT, TEL and TAN-strains. The western blot (right-hand side) indicates loss of acetylation on histone H4K4 and confirms loss of HAT3 function (Siegel *et al*., 2008). K4^{ac}, anti-H4K4^{acetylated}; K4^{un}, anti-H4K4^{unmodified}; WT, wild-type. An equivalent Coomassie-stained gel serves as a loading-control. (**B**) A PCR-based assay indicates disruption of native *SIR2rp1* and replacement with *NPT* and *BSD* selectable-marker cassettes in the INT, TEL and TAN-strains. (**C**) Southern blotting indicates DSB-repair in the INT-strain and in the derived *sir2rp1* and *hat3* null-strains. The loading control was prepared using a '7240' probe also from chr. 11 (see Materials & Methods). Other details are as for Figure 1C. (**D**) A PCR-based assay indicates a similar pattern of MMEJ in INT-cells and in the *sir2rp1* and *hat3* null-strains (see Glover *et al*, 2011). (**E**) A PCR-based assay indicates more rapid loss of the R^{s}P (*RFP:I-SceI:PAC)* 'DNA-break substrate' cassette following I-*Sce*I induction in the TAN^{hat3} strain.

Supplementary Table S1

**Oligonucleotide**	**Sequence**
hat3koF / H31	ccactagtaccccagtaga
hat3koR / H34	ctggtacctcagaaacagg
sir2rp1koF / 3C	ggcaagctgggtggcttt
sir2rp1koR / 5B	cccgtctcgtcattctctg
MMEJF / RFP5FU	gatc*aagctt*atggtgcgctcctccaag
MMEJR / Pac3Pol1	gatc*gctaga*tcaggcaccgggcttgc
4250F / 1.55F	gatcttaattaaatggtaggaggatatttacgt
4250R / 1.55R	gatctctagatcggcttagtttccgcac
SceJF	gcggatagggataacaggg
rad51tarN5F	gatc*gcggccgc*tgctacactactgctaccg
rad51tarX5R	gatc*tctaga*aagcaactccatagttttcc
rad51tarB3F	gatc*gggccc*acatgcctccactacacgg
rad51tarA3R	gatc*ggtacc*agcaaggcagatcaagtatc

Sequences of oligonucleotides used for cloning or PCR assays. Relevant restriction sites are indicated in italics.
